# Examining the associations between pain, function, and behavioral symptoms with engagement in meaningful activity among residents with dementia in assisted living

**DOI:** 10.1002/alz.086606

**Published:** 2025-01-09

**Authors:** Sarah D Holmes

**Affiliations:** ^1^ University of Maryland Baltimore, Baltimore, MD USA

## Abstract

**Background:**

Engagement in meaningful activity is beneficial for residents with dementia and is associated with improved quality of life. Meaningful activity refers to activities that provide meaning and value to the person and are tailored to individualized interests and preferences. The purpose of this study was to examine factors associated with engagement in meaningful activity among residents with dementia in assisted living. Specifically, it was hypothesized that function, pain, and behavioral symptoms would be associated with engagement in meaningful activity after controlling for residents’ age, gender, comorbidities, and cognition.

**Method:**

This study included baseline data collected from the randomized controlled trial, Meaningful Activities for Managing Behavioral Symptoms of Distress (MAC‐4‐BSD). Assisted living residents were eligible if they were ≥55 years old, live in a participating setting, and screen positive for dementia based on the Saint Louis University Mental Status exam (SLUMS) and AD8 Dementia Screening Interview. Residents were excluded if they were enrolled in hospice, admitted for short term respite, or had a significant neurological condition not related to ADRD. Residents’ engagement in meaningful activity was evaluated based on the Engagement in Meaningful Activities Survey (EMAS). Stepwise linear regression was used to examine resident factors associated with engagement in meaningful activity.

**Result:**

A total of 71 residents from 5 assisted living settings were included in the sample. Most participants were female (n = 52, 73%), White (n = 62, 87%), and mean age was 85 years old (SD = 8.2). Controlling for age, gender, comorbidities, and cognition, pain was significantly associated with engagement in meaningful activity (b = ‐2.1, p = .03). There were no associations between function and behavioral symptoms with engagement in meaningful activity (p>.05) and the model explained 6.1% of the variance in engagement in meaningful activity.

**Conclusion:**

Findings from this study offer insights into residents’ engagement in meaningful activity in assisted living. Ongoing research is needed to consider the impact of the physical and social environment and other possible factors that may also be associated with meaningful activity.

